# Towards national systems for continuous surveillance of antimicrobial resistance: Lessons from tuberculosis

**DOI:** 10.1371/journal.pmed.1002658

**Published:** 2018-09-14

**Authors:** Amitabh B. Suthar, Patrick K. Moonan, Heather L. Alexander

**Affiliations:** Center for Global Health, Centers for Disease Control and Prevention, Atlanta, Georgia, United States of America

## Abstract

In a Perspective on the research article from Jacobson and colleagues, Amitabh Suthar and colleagues from the Centers for Disease Control and Prevention discuss the importance of and considerations for developing real-time and large-scale reporting systems for tracking and controlling antimicrobial resistance.

## Antimicrobial resistance (AMR) surveillance

Drug resistance to microbes, including bacteria, viruses, fungi, and parasites, has reduced the treatment options for newly diagnosed infections [1]. The World Health Organization (WHO) recommends a core set of actions to control AMR, including surveillance to continuously monitor trends in AMR [2–4]. Surveillance is essential to (1) understand the prevalence and susceptibility patterns to inform national treatment guidelines, (2) understand individual-level transmission risk factors to reengineer health systems and community-based approaches, and (3) characterize population-level transmission networks to reduce emergence and spread of resistant organisms. To date, periodic surveys and studies have been widely used to generate these vital data.

In a recent *PLOS Medicine* research article, McIntosh and colleagues share South Africa’s experience in continuous surveillance for tuberculosis (TB) drug resistance [5]. Utilizing geospatial methods in Western Cape Province, McIntosh and colleagues were able to identify potential drug-resistant TB transmission “hotspots” for nearly 1 million TB cases and reveal increases and decreases in rifampicin-resistant TB in different locations. Although these data facilitated action at the subnational level, national-level decision-making requires nationally representative data. In order to use continuously generated data for national TB drug resistance surveillance, WHO recommends that at least 75% of new pulmonary TB cases nationally have drug susceptibility testing (DST) results for at least rifampicin [6]. Achieving availability of surveillance data for 75% of new pulmonary TB cases would require (1) having DST specimens collected in over 90% of detected pulmonary TB cases, (2) having required data elements accompany over 90% of DST specimens, and (3) having valid DST results in over 90% of DST specimens. Achieving availability of surveillance data for 75% of other pathogens may also be critical for national representativeness with other pathogens. In this Perspective, we describe considerations related to these areas for national AMR surveillance, drawing on the experience of McIntosh and colleagues.

## Patient coverage

Countries may need to consider various policies to ensure 90% of detected cases have DST at the national level. First, national diagnostic algorithms could systematically screen all cases for drug resistance. When diagnostic resistance assays are restricted to certain populations, such as those who may be at increased risk of resistance because of past treatment failure, the generalizability of the routinely generated data changes from representing the national disease cohort to specific subpopulations eligible for DST. Second, lists of nationally essential in vitro diagnostics are being developed and deployed and may affect DST technologies that are registered, centrally procured, and nationally distributed across laboratories and/or health facilities. Linking national diagnostic algorithms to essential diagnostic lists can facilitate national access to DST [7]. In the absence of this linkage, there could be discordance between national policy and access, which would introduce geographical biases in continuously generated data. Third, DST coverage within national health insurance benefit packages, as part of universal health coverage, may affect uptake. Specifically, if DST is not covered by benefit packages and requires out-of-pocket payments, vulnerable and poor populations could decline this service and introduce selection bias into continuously generated data.

WHO currently recommends universal DST in its End TB Strategy [8]. Unfortunately, in 2016, only 26 of 48 surveyed countries reported a national policy and algorithms indicating universal access to DST, and globally, only 41% of bacteriologically confirmed new and previously treated cases were tested for at least rifampicin resistance [9]. Upon identifying resistance to rifampicin, recommended diagnostic algorithms assess susceptibility to additional drugs to determine whether the TB strain is multidrug resistant (i.e., MDR TB) or extensively drug resistant (i.e., XDR TB) [6,10,11]. Since 2011, the South African TB diagnostic algorithm begins with smear and/or culture to confirm *Mycobacterium tuberculosis* complex; if positive, screening for rifampicin resistance is evaluated with Xpert MTB/RIF and is confirmed using line probe assay [5]. Information on out-of-pocket expenditures required for DSTs as part of this algorithm was not specified by McIntosh and colleagues. Nonetheless, overall, McIntosh and colleagues reported that 49% of confirmed TB cases were tested for rifampicin resistance [5]. Further expansion toward universal eligibility, access, and uptake of DST is key to maximizing patient coverage.

## Information completeness

National health information systems facilitate DST data collection, transmission, storage, and analysis. Communication between laboratory information systems and electronic health records helps ensure DST results are linked to an individual patient record. Unfortunately, recent studies have called into question the completeness and reliability of the laboratory and electronic surveillance systems. It is possible to learn from TB in South Africa [12–14]. McIntosh and colleagues leveraged the National Health Laboratory Services database in Western Cape Province [5]. Since this database does not contain unique patient identifiers, a deduplication algorithm utilizing name, surname, age, location, and sex was used to link specimen results to individual patients. In this study, 2.1% of specimens were removed from the South African analysis because an identifier or location was missing, and a further 15% of specimens were removed from geospatial analyses because the specimens were submitted from nonclinic locations [5]. Countries may need to determine the minimum data elements required to meet their pathogen’s surveillance objectives.

## Laboratory performance

Robust laboratory quality management systems are the cornerstone of accurate and reliable DST results. Such systems encompass the complete spectrum of diagnostic services from collection of specimens, storage in proper conditions, transportation to laboratories, laboratory testing, and result reporting. Recognizing the critical role of laboratory quality for appropriate patient care and disease surveillance efforts, WHO recommends that by 2020, all laboratories conducting TB testing are enrolled in external quality assessment (EQA) programs for all tests they conduct, all National Reference Laboratories are accredited to ISO 15189 standards, and sites performing DST are deemed proficient by EQA panel testing [15]. Developing national competencies, training the workforce to instill them, and monitoring the performance of laboratory systems are important means to achieve this target for TB and to achieve similar standards for other pathogens [15]. In recent years, a number of tools and resources have been developed to guide TB laboratory quality monitoring and improvement [16]. Enhanced global support and commitment, however, are required to sustain achievements to date and address noted weaknesses in DST laboratory performance to achieve WHO targets and ensure provision of accurate test results [17]. McIntosh and colleagues reported that 0.1% of specimens were excluded because they were indeterminate, suggesting that a high level of laboratory performance is possible.

## Surveillance approaches

Given the potential for selection, information, and measurement bias in continuous surveillance data systems, disease programs and countries will be left wondering whether they can make policy decisions on the basis of continuously generated data or whether surveys are also needed. Surveys can be robust and detailed and provide nationally representative data but can be logistically complex and expensive and require external technical support [6]. Many countries conduct nationally representative drug resistance surveys for TB [6]. South Africa is one country that is transitioning away from TB drug resistance surveys after national scale-up of DST [9,18]. The continuous TB drug resistance surveillance system in Western Cape produced a rifampicin resistance estimate similar to that generated in a recent national survey (4.6% versus 4.3%, respectively) [19]. However, only approximately 47% of diagnosed TB cases in the continuous system met the minimum requirements for analysis (i.e., specimen collected for DST, minimum data collected, and valid laboratory result available). If there are biases in the data from other provinces, ongoing surveillance in a sample of nationally representative sites (i.e., continuous sentinel surveillance) may be an interim solution to generate data while continuing to build a national system for continuous surveillance (Table 1).

**Table 1:** Comparison of surveys, continuous sentinel surveillance, and national systems for AMR surveillance.

	Objectives		Programmatic context		Resource requirements	
	Inform national guideline decisions	Can detect outbreaks	Requires DST to be clinically indicated	Requires national laboratory infrastructure for specimen collection, processing, transport, and storage	Additional training for facility and laboratory staff	Additional financing outside of routine program
Survey	Yes	Yes, at survey sites only if baseline incidence is known	No	No	Yes	Yes
Continuous sentinel surveillance	Depends on sampling approach for sentinel sites	At sentinel sites only	No	No	Initially (will reduce over time if same sites are utilized)	Yes
National system for continuous surveillance	If minimum requirements for 75% of detected cases are met	Yes	Yes	Yes	No	Limited

Abbreviations: AMR, antimicrobial resistance; DST, drug susceptibility testing.

## Future outlook

Standardized AMR tools can help low- and middle-income countries ascertain biases in different surveillance approaches and guide them to an appropriate selection based on their pathogen(s) of interest, objectives, and context. This guidance is available for TB [6]. On the other hand, not all surveillance approaches have been fully described for HIV, malaria, and priority bacterial pathogens [20–22]. DST in clinical care also plays a key role in system development, as routinely generated genotyping data can be leveraged for national systems for continuous surveillance when DST is clinically indicated. In addition to potentially informing national decisions, continuous surveillance has increased geographical granularity. McIntosh and colleagues were able to detect a resistance outbreak in a district and characterize districts with higher resistance due to poor health system access or higher migration of drug-resistant bacteria [5]. This novel use of spatial analysis helps local programs "see" where AMR resistance is accumulating and monitor trends over time, providing a potentially powerful tool to guide control and prevention efforts. This use of AMR surveillance data may need further encouragement and promotion. Moreover, routinely generated genotyping data have been used to characterize sources of transmission and guide prevention efforts [23]. Having these data available as an early warning could allow swift action and mitigate perpetuation of AMR strains. Therefore, regardless of whether a final policy decision is taken based on continuously generated data, more countries may find it worthwhile to explore the use of continuous AMR surveillance as part of national strategies to contain AMR.

## Abbreviations

AMR: antimicrobial resistance
DST: drug susceptibility testing
EQA: external quality assessment
MDR: multidrug resistant
TB: tuberculosis
WHO: World Health Organization
XDR: extensively drug resistant
